# Referral rates for children with acute gastroenteritis: a retrospective cohort study

**DOI:** 10.3399/bjgpopen20X101053

**Published:** 2020-07-22

**Authors:** Pien Ingrid Wolters, Gea Holtman, Freek Fickweiler, Irma Bonvanie, Anouk Weghorst, Johan Post, Boudewijn Kollen, Marjolein Berger

**Affiliations:** 1 Department of General Practice and Elderly Care Medicine, University Medical Centre Groningen, University of Groningen, Groningen, The Netherlands; 2 Department of Out-of-Hours Service Groningen, Groningen, The Netherlands

**Keywords:** After-hours care, gastroenteritis, pediatrics, paediatrics, primary care, referral

## Abstract

**Background:**

Hospital admission rates are increasing for children with acute gastroenteritis. However, it is unknown whether this increase is accompanied by an increase in referral rates from GPs due to increased workloads in primary care out-of-hours (OOH) services.

**Aim:**

To assess trends in referral rates from primary care OOH services to specialist emergency care for children presenting with acute gastroenteritis.

**Design & setting:**

This retrospective cohort study covered a period from September 2007–September 2014. Children aged 6 months to 6 years presenting with acute gastroenteritis to a primary care OOH service were included.

**Method:**

Pseudonymised data were obtained, and children were analysed overall and by age category. Χ^2^ trend tests were used to assess rates of acute gastroenteritis, referrals, face-to-face contacts, and oral rehydration therapy (ORT) prescriptions.

**Results:**

The data included 12 455 children (6517 boys), with a median age of 20.2 months (interquartile range [IQR] 11.6 to 36.0 months). Over 7 years, incidence rates of acute gastroenteritis decreased significantly, and face-to-face contact rates increased significantly (both, *P*<0.01). However, there was no significant trend for referral rates (*P* = 0.87) or prescription rates for ORT (*P* = 0.82). Subgroup analyses produced comparable results, although there was an increase in face-to-face contact rates for the older children.

**Conclusion:**

Incidence rates for childhood acute gastroenteritis presenting in OOH services decreased and referral rates did not increase significantly. These findings may be useful as a reference for the impact of new interventions for childhood acute gastroenteritis.

## How this fits in

Although it is known that hospital admission rates are increasing, the authors are not aware of research into the trends in referral rates for children with acute gastroenteritis presenting to primary care OOH services. The authors therefore investigated whether referral rates to specialist emergency care from a primary care OOH service increased over a 7-year period for children with acute gastroenteritis.

## Introduction

Most children that are younger than 5 years will suffer from at least one episode of acute gastroenteritis.^1^ Although these episodes are generally self-limiting and uncomplicated, they can lead to severe dehydration, particularly in young children.^2^ Over the decade from 1999–2010, hospital admission rates for acute gastroenteritis increased by 31% in England.^3^ This increase has not been associated with increased severity, with most cases being for short-term admissions (<1 day) that possibly could have been managed in primary care.^3^ High emergency admission rates are often thought to be inversely related to primary care quality, but, presumably, a complex interplay of factors is responsible for the observed increase in hospital admission rates.^4^

Primary care OOH services are regional centres in which multiple GPs work in shifts to cover patients outside of normal working hours.^5^ Patients in the Netherlands must go through triage by telephone before they are invited for face-to-face contact with a GP in the OOH service. Factors thought to have influenced the increase in hospital admission rates include complicated access to the OOH service, loss of continuity in GP care, a drive for shorter hospital stays (also leading to increased readmission rates), the impact of social media, and the expectations of parents and professionals for the treatment of a sick child.^4^ In addition, GPs are experiencing high workloads in OOH services,^6^ which may be due to inaccurate triage of children by telephone assistants. In turn, this may contribute to more referrals to paediatric emergency departments and consequent hospital admissions for children who could be better managed at home with ORT.^7^

Although trends in hospital admission rates are known, the authors are not aware of research into the trends in referral rates for children with acute gastroenteritis presenting to primary care OOH services. The authors therefore investigated whether referral rates to paediatric emergency care from a primary care OOH service increased over a 7-year period for children with acute gastroenteritis. In addition, factors potentially related to that trend were explored, focusing on rates of the incidence of acute gastroenteritis, face-to-face contacts, and ORT prescriptions.

## Method

### Study design

This retrospective cohort study was performed using information obtained between September 2007–September 2014. Data for children aged 6 months to 6 years were obtained from the electronic database of a primary care OOH service. The primary outcome was the referral rate from this service to secondary care.

### Setting and triage procedure

Pseudonymised data were obtained from the electronic database of a primary care OOH service that included 290 collaborating GPs providing care for approximately 650 000 residents in the north of the Netherlands.^8^ Triage was initially performed over the telephone by trained assistants who assessed the urgency of a consultation based on the guidelines of the Dutch College of General Practitioners. They were then able to offer advice over the telephone — including advice to administer ORT — or make an appointment for face-to-face contact with a GP.^9^ If the patient was seen by a GP in a face-to-face contact, the GP decided if referral was necessary or if the patient could be managed at home. The assistant and GP record their findings in the patient’s medical record, which contains information on the contact date, demographics, symptoms, physical examination, additional testing, diagnosis, prescriptions, and referrals.

### Study population and contact selection

The study included children aged 6 months to 6 years who were diagnosed with acute gastroenteritis and seen in the OOH service during the study period. First, all contacts of children aged 6 months to 6 years were selected, and their medical records were extracted and saved in a database. All patient information was pseudonymised by the OOH service. Second, a computer search was performed to select all contacts with the words ‘*diarrhoea*’ and/or ‘*vomiting*’ (or synonyms of these words) in the history record. The results were checked for false negatives by randomly extracting 10% of all OOH service contacts over the study period (*n* = 5000) and hand checking if any children with diarrhoea and/or vomiting had been missed. The computerised search was then adapted, and the false negative screening was repeated until no eligible contacts were missed. Three researchers (two medical students and a GP) also hand searched all contacts in which the child presented with diarrhoea and/or vomiting to exclude those with chronic diarrhoea (that is to say, those with symptoms for ≥2 weeks).

The study defined a diagnosis of acute gastroenteritis as follows: 1) a registered diagnosis of ‘*gastroenteritis*’, or synonyms; or 2) a registered diagnosis of ‘*viral infection*’ or ‘*vomiting*’ if diarrhoea was a presenting symptom; or 3) if no diagnosis was recorded, but diarrhoea or vomiting was the presenting symptom and other plausible causes were not mentioned. Contact selection was performed by three researchers, and any uncertainties were discussed with an expert panel (two GPs). If children contacted more than once within two weeks, it was counted as one episode. If children contacted more than once, but with an intervening period of more than 2 weeks, this was counted as separate episodes.

### Data extraction

The following data were extracted by three researchers using a structured form: contact date, contact type (telephone, face-to-face), age, symptoms and signs, referral, and any medication or ORT prescribed (or self-prescribed). Before starting full data extraction, a pilot was performed to determine the level of agreement between researchers in the extracted data (Cronbach’s alpha, ≥0.87). After a consensus meeting, agreement was retested for a random sample of 10% of all of the included contacts (Cronbach alpha, ≥0.85). Thus, there was a good level of agreement in the information extracted between researchers.

### Outcomes

The primary outcome was the referral rate, with the total number of contacts per year as the denominator. Secondary outcomes were to analyse the incidence rate of acute gastroenteritis, the rate of each contact type (for example, face-to-face or telephone), and the rate of ORT prescriptions.

### Statistical analysis

Descriptive data are reported as medians and IQRs, or as numbers and percentages. Trends were evaluated for all primary and secondary outcomes. In addition, subgroup analyses were performed for age categories of 6 to 12 months and 1 to 6 years. All trend analyses were conducted using the Χ^2^ test (two-sided), and were considered significant if *P*<0.05. Data were analysed using IBM SPSS (version 25.0).

## Results

### Patient characteristics

**Figure 1.**
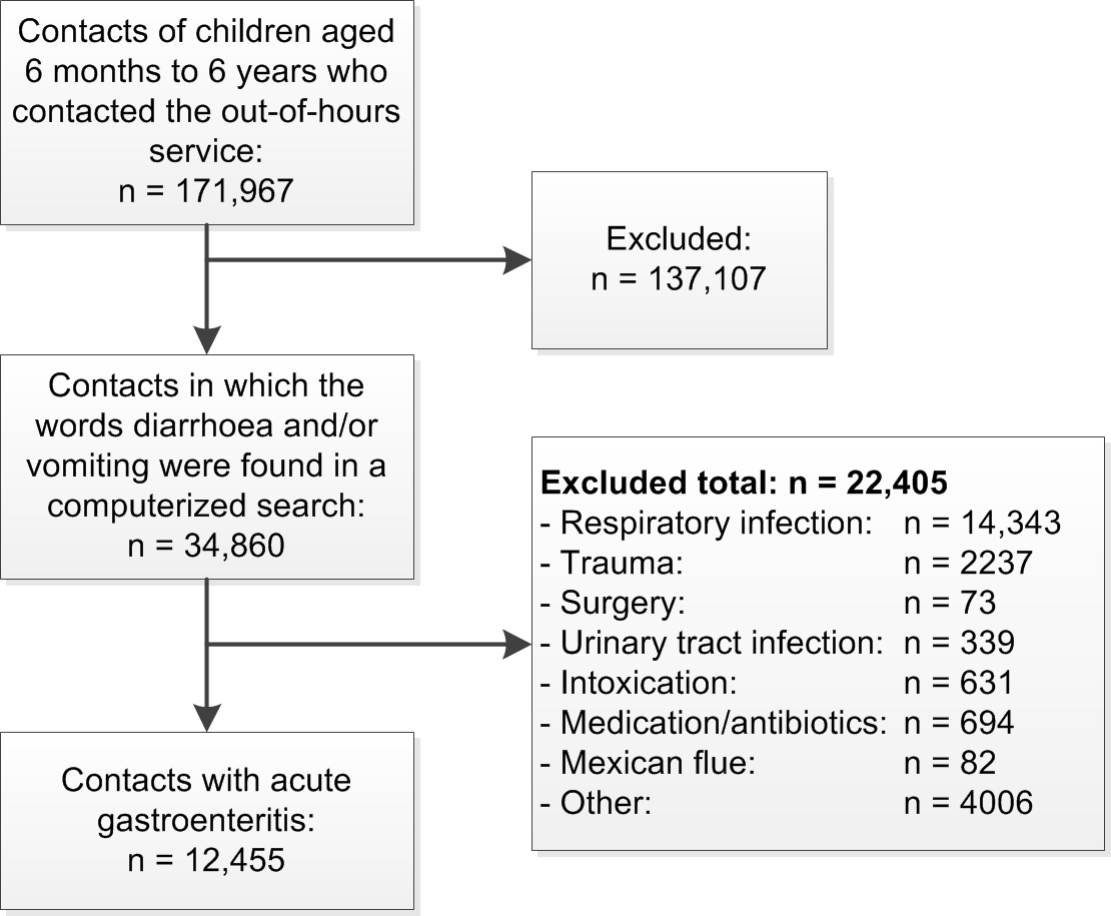
Flowchart of patient selection

In total, 171 967 contacts with the OOH service were recorded for children aged 6 months to 6 years during the study period. Among these, 34 860 were for diarrhoea and/or vomiting, and a subset of 12 455 (9432 children) were diagnosed with acute gastroenteritis (Figure 1). For those with acute gastroenteritis, multiple contacts were recorded in 3023 cases (specifically, two times for 1613 children, three times for 396 children, four times for 112 children, five times for 45 children, six times for 13 children, seven times for five children, and eight times for one child).

The median age was 20.2 months (IQR 11.6 to 36.0), and boys accounted for 6517 contacts (52.3%). Regarding presentation, 2678 (21.5%) contacts had only diarrhoea, 3934 (31.6%) had only vomiting, and 5843 (46.9%) had both diarrhoea and vomiting. In total 9777 (78.5%) contacts presented with vomiting and 6614 (53.1%) with diarrhoea (Table 1). For the 1036 contacts (8.3%) referred with acute gastroenteritis, age and sex were comparable to those in the overall cohort, but a higher proportion had both diarrhoea and vomiting.

**Table 1.** Characteristics of Dutch children aged 6 months to 6 years with acute gastroenteritis who were seen in a primary care out-of-hours service (2007–2014)

**Characteristics**	**Total (*n* = 12 455**)	**Referred children (*n* = 1036**)
Male sex, *n* (%)	6517 (52.3)	556 (53.7)
Median age, months (IQR)	20.2 (11.6 to 36.0)	18.0 (12.0 to 32.0)
**Age categories**		
6 months to <1 year, *n* (%)	3229 (25.9)	249 (24.0)
1 to 6 years, *n* (%)	9226 (74.1)	787 (76.0)
**Presenting symptoms**		
Diarrhoea only, *n* (%)	2678 (21.5)^a^	106 (10.2)^b^
Vomiting only, *n* (%)	3934 (31.6)^c^	214 (20.7)^d^
Diarrhoea and vomiting, *n* (%)	5843 (46.9)	716 (69.1)

a No information about vomiting in patient record (*n* = 1621).

b No information about vomiting in patient record (*n* = 73).

c No information about diarrhoea in patient record (*n* = 2006).

d No information about diarrhoea in patient record (*n* = 112).

IQR interquartile range

### Trend analyses

Table 2 presents the results of the trend analyses overall and for the two age subgroups over the 7-year study period. In both the overall and subgroup analyses, no significant increase in the trend for referral rates was found (overall median 8.1% ). However, there was an increasing trend in face-to-face contact rates for all children with acute gastroenteritis (*P*<0.01). Subgroup analyses confirmed that this increasing trend was only statistically significant for children aged 1–6 years (*P*<0.01). ORT prescription rates did not change significantly (*P* = 0.82). Finally, there was a significantly decreasing trend in the incidence rate of acute gastroenteritis presenting to the OOH service in both the overall and the subgroup analyses (*P*<0.01).

**Table 2.** Trend analyses for Dutch children aged 6 months to 6 years with acute gastroenteritis seen in a primary care OOH service (2007 to 2014), grouped by the total cohort (*n* = 12 455), age <1 year (n = 3229), and age ≥1 year (n = 9226)

	**2007/2008**	**2008/2009**	**2009/2010**	**2010/2011**	**2011/2012**	**2012/2013**	**2013/2014**	**Total**	***P* value**
**Total number of contacts** ^a^	24 920	25 088	27 772	24 466	24 459	22 923	22 339	171 967	–
**6 months to 6 years, *n* (%)** ^b^(*n* = 12 455)	2298 (9.2)	1994 (7.9)	1902 (6.8)	1648 (6.7)	1612 (6.6)	1784 (7.8)	1217 (5.4)	12455	<0.01
Referred, *n* (%)^c^	195 (8.5)	162 (8.1)	147 (7.7)	154 (9.3)	127 (7.9)	163 (9.1)	88 (7.2)	1036	0.87
Face-to-face contact, *n* (%)^c^	1186 (51.6)	1025 (51.4)	927 (48.7)	885 (53.7)	809 (50.2)	994 (55.7)	672 (55.2)	6498	<0.01
ORT prescription, *n* (%)^c^	533 (23.2)	415 (20.8)	424 (22.3)	343 (20.8)	367 (22.8)	432 (24.2)	242 (19.9)	2756	0.82
**Subgroup analyses**									
**6 months to <1 year, *n* (%)** ^b^(*n* = 3229)	545 (2.2)	554 (2.2)	568 (2.0)	438 (1.8)	413 (1.7)	416 (1.8)	295 (1.3)	3229	<0.01
Referred, *n* (%)^c^	33 (6.1)	52 (9.4)	42 (7.4)	47 (10.7)	30 (7.3)	21 (5.0)	24 (8.1)	249	0.73
Face-to-face contact, *n* (%)^c^	297 (54.5)	298 (53.8)	274 (48.2)	243 (55.5)	233 (56.4)	231 (55.5)	172 (58.3)	1748	0.10
ORT prescription, *n* (%)^c^	135 (24.8)	129 (23.3)	128 (22.5)	91 (20.8)	109 (26.4)	99 (23.8)	61 (20.7)	752	0.58
**1** **year** **to** **6** **years** **,** ***n*** **(%)** ^b^(*n* = 9226)	1753 (7.0)	1440 (5.7)	1334 (4.8)	1210 (4.9)	1199 (4.9)	1368 (6.0)	922 (4.1)	9226	<0.01
Referred, *n* (%)^c^	162 (9.2)	110 (7.6)	105 (7.9)	107 (8.8)	97 (8.1)	142 (10.4)	64 (6.9)	787	0.98
Face-to-face contact, *n* (%)^c^	889 (50.7)	727 (50.5)	653 (49.0)	642 (53.1)	576 (48.0)	763 (55.8)	500 (54.2)	4750	<0.01
ORT prescription, *n* (%)^c^	398 (22.7)	286 (19.9)	296 (22.2)	252 (20.8)	258 (21.5)	333 (24.3)	181 (19.6)	2004	0.93

^a^Total number of contacts including all children aged 6 months to 6 years visiting the OOH service. ^b^Denominator is total number of contacts 6 months to 6 years. ^c^Denominator is total number of contacts with acute gastroenteritis in each specific age category.

OOH out-of-hours ORT oral rehydration therapy

## Discussion

### Summary

This study gives important insights into referral rates for childhood acute gastroenteritis from a primary care OOH service to paediatric specialist care between 2007 and 2014, a period during which there was no change in guidelines. Incidence rates for childhood acute gastroenteritis decreased and this study could not show a trend in referral rates in both the overall and subgroup analyses. The median referral rate was 8.1%. The study found a statistically significant increasing trend in face-to-face contact rates. This was mainly due to a significant increasing trend in face-to-face contact rates in children aged 1–6 years. Referral was more likely for children reporting both diarrhoea and vomiting, and almost one in five children received advice or a prescription for ORT.

### Strengths and limitations

The main strength was the inclusion of a relatively large number of patient contacts. Data were then obtained in a structured manner with good reliability among the raters and discussion of doubtful contacts. Missing data were also minimised because Dutch law (The Medical Treatment Agreement Act) requires that information on referrals and prescriptions be recorded. Moreover, when the authors screened a random sample for false negatives, they confirmed that few children with diarrhoea and/or vomiting were missed by the computerised selection method.

Some limitations do need to be considered, such as the decision to include only those aged 6 months to 6 years, and to perform subgroup analysis at a cut-off of 1 year. The overall age range was chosen because it corresponded to the peak incidence of acute gastroenteritis^10^ and the group that most often contacts primary care OOH services.^5^ Younger children were excluded because they are at increased risk of dehydration, meaning that any referral decisions may only reflect age.^11^ In the subgroup analyses, age groups were predefined based on their assumed risk for a complicated course. However, this age cut-off was arbitrary, and it may have been preferable to use the 2 year cut-off advised in the 2014 revision of Dutch guidelines.^10^ Furthermore, multiple contacts were recorded in 3023 cases, which might have influenced the magnitude of the referral rate.

The health care system in the Netherlands is comparable to those in Denmark, Sweden, the UK, Australia, and New Zealand, which are based on the GP serving as a gatekeeper to further care.^12^ However, watchful waiting is a common strategy in the Netherlands, with emphasis on telephone advice and relatively few people getting face-to-face contact with the GP.^13^ For example, 5% and 22% of community cases visit their GP because of acute gastroenteritis in the Netherlands and New Zealand, respectively.^14^ Therefore, trends in referral rates in the Netherlands could also differ from those in other countries.

### Comparison with existing literature

It was notable that there was no statistically significant increase in referral rates from the OOH service to the emergency department, which ran counter to the authors' expectation based on a previous report on increasing hospital admission rates.^3^ The findings may indicate that parents attend the paediatric emergency department directly, possibly because of easier access to advice without the need for telephone triage.^4^ This could account for the increase in hospital admission rates of children that could be managed in primary care, despite referral rates from OOH services remaining stable. However, this does not seem a plausible explanation. Given that prognosis was worse among those self-referred with fever, parents appear to be capable of accurately evaluating the severity of illness and need for emergency paediatric care.^15^

The increasing trend in face-to-face contact rates for acute gastroenteritis in children was consistent with the findings of a Dutch study showing a similar increase in face-to-face contact rates to OOH services for other problems between 2009 and 2016.^6^ This may indicate a change in telephone triage practices at OOH services. In the Netherlands, most OOH services use a validated standard for triage to increase its efficiency and patient safety.^9^ However, unknown patients, anxious parents, high work pressures,^16^ and differing views of disease and illness can make triage challenging. Furthermore, it has been shown that telephone triage may be especially suboptimal for children with gastrointestinal complaints.^17^ These challenges may be associated with the increase in face-to-face contact rates for children with benign prognoses. This in turn, may contribute to the high work pressure experienced by GPs in these services, even while the absolute number of children presenting with acute gastroenteritis decreases.^6^

Subgroup analyses showed that the increase in face-to-face contact rates was only significant for children aged 1–6 years, in whom the risks of complications were low to moderate. It is unlikely that risk actually increased over time to justify this change, indicating that more children with a benign prognosis were allowed through to face-to-face contacts. Indeed, despite the increase in face-to-face contact rates, secondary triage by GPs did not result in a corresponding increase in referral rates. The likelihood of referral was increased if the child had an increased risk of dehydration, with referral rates being highest for those presenting with both vomiting and diarrhoea. This finding indicates that the quality of GP triage remained appropriate.

ORT is the recommended first line treatment for children at risk of dehydration or with mild, moderate, or severe dehydration, with proven efficacy.^2^ In the present study, the change in ORT prescribing rates was statistically insignificant, with approximately 22% receiving advice or a prescription, and non-prescribing justified by the presence of vomiting in about 80% of patients.^7^ This is consistent with the justifiable fear that vomiting will hamper the intake of ORT and may affect compliance. In referred children, ondansetron is an effective antiemetic, which could increase ORT uptake and compliance.^18^ As such, this medication may have a role in primary care, with the potential to prevent referrals to secondary care and manage patients in primary care.

The incidence rate of acute gastroenteritis in children almost halved during the study period (Table 2). However, this finding should be interpreted with caution because the annual incidence fluctuates widely for a range of reasons, with each of these having the potential to explain the observed variations.^19^ Moreover, only the first and last years of this study showed markedly different incidence rates, with relative stability observed in the intervening period. An explanation for the lower incidence and referral rates for acute gastroenteritis in 2013–2014 could be the lower reported incidence of rotavirus infections in that year.^20^ Given that rotavirus is known to be associated with a particularly complicated course,^21^ a lower incidence could be associated with fewer contacts and referrals.

### Implications for research and practice

These results showed that the trend in referral rates to secondary care is not significant. There are five aspects in the management of children with acute gastroenteritis that could potentially affect these rates in the future. First, the introduction of point-of-care tests for pathogens of acute gastroenteritis may affect management. Triaging children with gastrointestinal complaints based on their clinical signs and symptoms is challenging. It is therefore unsurprising that GPs have difficulties in distinguishing between children who will have uncomplicated courses and those who will have complicated courses requiring referral. Current guidelines do not recommend stool microbiological investigation for acute gastroenteritis in children.^10,11^ Research could therefore evaluate if specific pathogens are associated with a more severe course of acute gastroenteritis, and if demonstrated, should assess the added value of point-of-care tests in daily practice.

Second, rotavirus is the most common pathogen among children presenting with acute gastroenteritis in primary care,^10^ yet children had not been vaccinated against it during the study period. Implementing this vaccination in the future will influence the risk of a complicated course in children with acute gastroenteritis. This will influence the need for referral.

Third, although referral rates remained constant at a median of 8.1%, the percentage that was subsequently admitted to hospital was unknown. It would be interesting to know whether treating vomiting specifically could facilitate greater ORT intake in primary care, and thereby decrease referral rates. Ondansetron is often used with good efficacy as an antiemetic, and to increase ORT uptake and compliance in paediatrics.^18^ For now, ondansetron has only been shown to have benefit in hospital settings, at the more severe end of the spectrum, especially in children who are deemed unsuitable for discharge from emergency department. It might be argued that ondansetron may not be warranted, safe, or cost-efficient in children presenting to primary care. Concerns about diarrhoea, prolongation of QT-interval on electrocardiogram, and prescribing for minimal clinical benefit may challenge uptake in primary care. A study addressing the impact of ondansetron in primary care, focusing on children with acute gastroenteritis and prominent vomiting, is therefore highly needed.

A fourth management aspect is that not enough is known about the adherence to prescriptions for ORT. The presence of vomiting in around 80% of contacts in this study could result in poor compliance with ORT, or to GPs fearing poor compliance. Further research into ORT adherence, including qualitative research into the barriers to adherence, is therefore warranted.

Finally, the reasons for the increase in face-to-face contact by older children requires further research. Important questions in this research will include the reasons for parents contacting OOH services, the validity of telephone triage, and the availability of adequate and appropriate information about when parents should contact the OOH service.

In a 7-year period from 2007 to 2014, incidence rates for childhood acute gastroenteritis presenting at OOH services decreased, and referral rates remained stable. These findings may be useful as a reference against which the impact of new interventions for childhood acute gastroenteritis can be measured.
